# Electronic cigarettes: patterns of use, health effects, use in smoking cessation and regulatory issues

**DOI:** 10.1186/1617-9625-12-21

**Published:** 2014-12-15

**Authors:** Muhammad Aziz Rahman, Nicholas Hann, Andrew Wilson, Linda Worrall-Carter

**Affiliations:** St Vincent’s Centre for Nursing Research (SVCNR), Australian Catholic University, Melbourne, Australia; The Cardiovascular Research Centre (CvRC), Australian Catholic University, Melbourne, Australia; The University of Melbourne, Melbourne, Australia; St Vincent’s Hospital, Melbourne, Australia

**Keywords:** E-cigarettes, Electronic cigarettes, Smoking, Smoking cessation, Tobacco

## Abstract

**Background:**

Electronic cigarettes (e-cigarettes) are battery-powered devices that vaporize a liquid solution to deliver a dose of inhaled nicotine to the user. There is ongoing debate regarding their regulation.

**Objectives:**

This comprehensive narrative review aimed to discuss key issues including usage patterns, health effects, efficacy in smoking cessation and regulatory concerns with a view to informing future regulation and research agendas.

**Methods:**

PubMed, Scopus and Web of Science databases were searched using the terms (electronic cigarettes OR e-cigarettes) for articles in English, relevant to humans and published during January 2009-January 2014.

**Results:**

The literature search revealed 37 relevant articles. Findings suggest that e-cigarettes are mostly used by middle-aged current smokers, particularly males, to help them for quitting or for recreation. E-cigarettes contain very low levels of multiple toxic substances such as formaldehyde and acrolein, but these levels are many times lower than those found in cigarettes. They were found to have effectiveness in aiding smoking cessation to a limited degree. Debate continues regarding regulating their use for cessation versus heavy restrictions to control recreational use on the basis that it perpetuates nicotine addiction.

**Conclusions:**

The cytotoxicity and long term health effects of e-cigarettes are unknown. Nevertheless the e-cigarette market continues to expand, largely driven by middle-aged smokers who claim to be using e-cigarettes in an attempt to reduce or quit smoking. E-cigarettes may have some potential as smoking cessation aids and, in the researchers’ view, should therefore be subject to further research and regulation similar to other nicotine replacement therapies.

## Background

**Figure 1:**
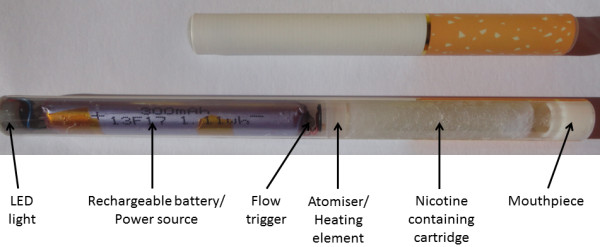
**Components of an electronic cigarette.**

E-cigarettes are battery-powered cigarette-shaped devices that vaporize a liquid solution which is inhaled orally to deliver a dose of nicotine to the user. The liquid solution, contained in a cylindrical cartridge, generally consists of propylene glycol in which nicotine and other aromas may be dissolved (Figure 1). The solution is vaporized when the user puffs on the device, activating a battery-powered heating element [1, 2]. The devices are available both in a cigarette-shaped form and newer ‘tank’ form which replaces the cylindrical cartridge with a larger solution tank, allowing the user to refill less frequently [3]. As tobacco leaves are not combusted in this process, manufacturers claim the resulting vapor is free of the 4000 toxic chemicals and carcinogens known to be produced by combustion in cigarettes [4].

E-cigarettes were invented by Chinese pharmacist Hon Lik in 2003, and subsequently became available globally, entering the European (EU) and American (US) markets in 2006 and 2007 respectively [1, 5]. Their use has grown rapidly; Google searches for ‘e-cigarettes’ increased by 5000% during 2011-12, 18% of US smokers have tried them and as of 2013 the industry is worth $2 billion in the US [6, 7]. This market growth in use is due in part to the implementation of novel marketing campaigns by e-cigarette manufacturers, including large tobacco companies that own some e-cigarette producers [7]. Indeed, in absence of market regulation, $15.7 million was spent on e-cigarette advertising by US manufacturers in the first quarter of 2013 alone [7].

There is much debate about the potential of e-cigarettes in various capacities, but given the relative paucity of scientific research investigating their safety and efficacy, they present a pressing dilemma to regulatory authorities. On the one hand, they have the potential to become a valuable smoking cessation aid and contribute to the momentum of existing tobacco control programs [8]. One study showed them to be as effective as nicotine patches in helping smokers to quit and superior to nicotine patches in reducing the number of cigarettes individuals smoked [9]. Conversely, there is concern that as long as they are unregulated, e-cigarettes may serve to re-normalize and re-glamorize smoking to vulnerable youth and developing world populations, thereby undermining the success of tobacco control activities [10].

Current debate about the devices is focused on their regulation, with a decision having been made in the EU and regulations set to be decided on in the US in mid-2014. Regulatory decisions will closely dictate the trajectory of the e-cigarette, both as a tool in tobacco harm reduction strategies and as a commercial product [6, 11]. Public health researchers favoring the devices’ role in smoking cessation are arguing for a compromise of measured regulation, at least at the outset, so as not to regulate the products out of existence [11]. Proponents of this argument cite unexpected consequences of existing laws on nicotine replacement products which they argue make them so restricted and unappealing that they act as a disincentive to quit smoking [11]. On the other hand, researchers that are skeptical of the devices’ potential role in tobacco harm reduction are arguing for strict regulation. They liken e-cigarettes to filtered and light cigarettes which were marketed as harm reduction strategies, but actually acted as disincentives to quitting and rather augmented tobacco use [6].

The objective of this paper is to provide a comprehensive narrative review of the existing literature pertaining to e-cigarettes, including usage patterns, motivations for use, health effects, potential role in smoking cessation and a summary of the current regulatory debate. By collating the findings of key research in these areas, this paper aims to inform regulatory decisions and highlight areas for further investigation.

### The device

Figure 1 illustrates the components of a standard e-cigarette. When the user inhales, airflow is created which activates the flow trigger. Then the LED light turns on and the heating element vaporizes the solution in the cartridge (which contains propylene glycol, nicotine and sometimes flavorings) into mist. The nicotine containing mist is then inhaled by the user [15]. Refill solutions in nicotine cartridges have been shown to contain low levels of several toxic substances [12–14]. These substances include carbonyl compounds, volatile organic compounds, nitrosamines, ultrafine particulate matter and heavy metals [4, 12–14]. These substances are of interest because they are known to be implicated in various disease processes. For example, carbonyl compounds detected include formaldehyde and acetaldehyde (known to be carcinogenic) and acrolein (implicated in the pathogenesis of cardiovascular diseases) [12]. Propylene glycol acts as the humectants in most refill solutions and whilst it is not cytotoxic in its liquid form, it has been found to exacerbate allergic respiratory symptoms including rhinitis and asthma, and the safety of inhaling its vaporized form, especially in the long-term, has not been tested in humans [13, 15].

## Methods

PubMed, Scopus and Web of Science databases were selected as the primary databases. We used the following search terms: (electronic cigarettes OR e-cigarettes). Fields were limited to ‘title and abstract’ for PubMed with ‘keywords’ also included for Scopus. We used 'topic' field for Web of Science searches. Searches were limited to English language, humans and published in the five years from January 2009 to January 2014. Our objective was to find articles on e-cigarettes which focused on the aims of this paper. Inclusion criteria were: articles focused on e-cigarettes specifically (not other smoking cessation therapies) and focused on any of the five key themes (usage patterns, motivations for use, health effects, potential role in smoking cessation and the current regulatory debate) according to the objective of this manuscript. Firstly, titles and abstracts were assessed, and then articles were included or excluded based on their relevance. The literature search revealed 37 relevant articles. Each of the articles was reviewed in details and summarized according to our five themes.

## Review

### Patterns of use

Available research on patterns of use is limited; some researchers quantify the amount of use, e.g. daily, occasional or experimental use, whereas others merely measure ever- versus never-use, which provides less robust information on usage patterns. Of the nine reviewed studies that investigated patterns of use, four quantified use by distinguishing among daily, occasional and experimental use, which is important to consider when discussing their findings [3, 16–18].

Six of the seven studies investigating smoking status found that e-cigarette use was more prevalent among current and former smokers than among never smokers [3, 17, 19–23]. In one study, 21% of current smokers were using e-cigarettes compared with 7% of former smokers and 1% of never smokers [20]. Although that does not rule out their role as bridging products for current smokers, it tends to discount the notion that the devices are being used by large numbers of never smokers as gateway products to further nicotine and tobacco use [24, 25]. The one exception to this trend is the study by Sutfin et al. which investigated e-cigarette use among university students [23]. Although ever-use of e-cigarettes among the sample of 4,444 students was low (4.9%), 12% of those e-cigarette users were never smokers [23]. Dutra et al also showed that e-cigarette use was associated with lower odds of abstinence from cigarette smoking among a large population of US school students [16].

There is reasonable consensus in the literature regarding the age profile of e-cigarette users, indicating that most users are current smokers. Seven studies investigated age and all found that use of e-cigarettes increased significantly during the third to fifth decades of life, and then declined [3, 17, 19–23]. Three studies reported the median age of e-cigarette use between 40 and 50 years [3, 17, 18]. Studies investigating use among young people reported variable rates of use among adolescents [19, 22, 26, 27] with two studies reported rates less than 1% [19, 22]. However, Lee et al. found that 9% of Korean adolescents ever used e-cigarettes and 5% used within last month [27]. Furthermore, Goniewicz et al found that almost one in four (24%) Polish high school students aged 15-19 years had tried e-cigarettes and 8% had done so within last month [26]. It also needs to be acknowledged that there is lack of most recent data on e-cigarette use among adolescents and it is unknown whether popularity of e-cigarettes has increased in this group.

Two studies assessed demographic variables and reported similar findings in terms of gender, socioeconomic status and geographical distribution of e-cigarette users [20, 23]. Two studies found that use was significantly more common among males, but that there was no significant difference in use according to the level of education or income [3, 17]. Studies suggest that e-cigarette use is concentrated in Europe and the US, with a small but significant level in Brazil [3]. Within the US, there does not appear to be any significant difference in use according to geographical location [17].

### Motivation for use

There is conjecture about consumers’ motivation for using e-cigarettes, with one body of research suggesting people are using them to quit smoking [3, 17, 18, 28], while another expresses concern that a great deal of e-cigarette use is recreational rather than quit-related [21, 23].

Several studies demonstrated the recreational element of e-cigarette use. In one study that included two surveys of more than 3500 e-cigarette users, only one showed a marginally significant correlation between use and a quit attempt in the last three months [21]. Sutfin et al. studied university students (42% of whom were current smokers) and found no established association between e-cigarette use and intention to quit smoking [23]. Furthermore, that study also found that a group of current smokers (42%) perceived that e-cigarettes was not less harmful than tobacco cigarettes. Dawkins et al also found that motivations for using e-cigarettes were mixed [3]. Four in ten current smokers (40%) cited a desire for a ‘partial alternative’ to smoking as a reason for using e-cigarettes, while 66% stated that they wanted a ‘complete alternative’ , again suggesting that whilst most use is quit-related, a substantial proportion is recreational.

### Health effects

The advent and rapid uptake of e-cigarettes has spawned a number of well-placed questions regarding their safety, which include queries as to the toxicity of their refill fluids and of vapor, as well as their potential to cause cancer or affect other organ systems beyond the lung.

Eight of the reviewed studies analyzed the health effects of e-cigarettes and two of those measured toxin levels in the vapor. Both detected toxins in vapor, but at levels which were either dramatically lower than those found in tobacco cigarettes or clinically insignificant [12, 13]. Goniewicz et al. found that the vapor of all 12 e-cigarette brands tested contained at least 9 of the 11 toxic substances measured. When compared to toxicity levels known to be present in tobacco smoke, concentrations of substances in e-cigarette vapor were dramatically lower in e-cigarettes. Levels of nitrosamines were 380-fold lower and acetaldehyde were 450-fold lower in e-cigarettes than conventional tobacco cigarettes. Levels of carcinogenic formaldehyde were only 9-fold lower in e-cigarettes compared to tobacco cigarettes, which the researchers deemed ‘comparable’ to cigarette smoke [12]. The other study looking at toxins in vapor was funded in most part by the American National Vapers’ Club (a possible conflict of interest) also found the vapor of all 4 brands tested contained at least 5 of the 7 toxic chemicals measured. However, levels of these substances were deemed to be clinically insignificant and led to ‘no discernible health impacts’ of the endpoints investigated.

Neither study investigating the effects of e-cigarettes on lung function suggested that they had any adverse effects [29, 30]. The study by Flouris et al. found no degree of airflow obstruction, as measured by FEV_1_/FVC ratio, after both active and passive exposure to e-cigarette vapor [30]. The other study found that smoking an e-cigarette causes no airway obstruction, whereas cigarette smoking causes mild airway obstruction and a small increase in lung inflammation that lasts less than an hour [29]. Conversely, a study by Vardavas et al found that airway resistance and airway impedance increased among a group of e-cigarette users compared with a control group after 5 minutes of use, while the fraction of expired nitrous oxide also declined in that group compared to controls [31]. The fraction of expired nitrous oxide is important because it is implicated in the pathophysiology of airways diseases associated with smoking, and so lower expired levels imply higher residual levels in the airways to promote this pathogenesis. Although those changes were statistically significant, they only measured short-term parameters which were not clinically significant [31].

There is conjecture within a limited body of research regarding the cytotoxicity and cancer-causing ability of e-cigarette refill solutions and vapor. One study, by Bahl et al, showed some e-cigarette refill solutions to be either moderately or highly cytotoxic to a variety of cell types [32]. Embryonic and newborn stem cells were found to be more sensitive to those cytotoxic effects than differentiated adult fibroblasts, to a degree that researchers deemed would be enough to cause embryonic loss or developmental defects in pregnancy [32]. Consistent with findings in other studies of toxic substances in refill solutions, those results were deemed not to be due to nicotine or humectant content, but rather were correlated with the variety and concentrations of chemicals that flavor the solutions [32]. Contrary to those findings, a study by Romagna et al showed that e-cigarette vapor was not cytotoxic [4]. However, that study was funded by an e-cigarette manufacturer to test its own products, making the results highly questionable.

### Smoking cessation

There are two key issues to assess with regard to smoking cessation. Firstly, whether people are using e-cigarettes to quit smoking, and secondly whether they are effective for that purpose. As foreshadowed, it has already been established that some e-cigarette use is recreational [17, 33], but a larger proportion of the users are inhaling with an intention to quit smoking. Two studies reported the proportion of users attempting to quit, which ranged from 60% to 76% [3, 17]. Among those intending to quit smoking, only very small numbers of participants claimed to be using the devices as an alternative in settings where smoking is banned [3, 15, 24].

All of the studies investigating e-cigarettes for smoking cessation have demonstrated they may be effective both in aiding quit attempts and reducing the number of cigarettes smoked [3, 9, 17, 18, 28]. Two randomized controlled trials (RCTs) demonstrated favorable quit rates among users of nicotine-containing e-cigarettes when compared to either placebo or other nicotine replacement therapies (NRT) [9, 28]. One RCT found that 11% participants achieved abstinence from tobacco smoking at 12 months using nicotine e-cigarettes compared to 4% participants who used placebo; while the other RCT found that 7% participants achieved complete abstinence at 6 months compared to 6% participants using nicotine patches and 4% participants with placebo devices. In one study, abstinence was defined as complete self-reported abstinence from tobacco smoking - not even a puff - together with an exhaled carbon monoxide (eCO) concentration of ≤7 ppm [28]. In the other study, abstinence was defined as self-reported abstinence over the whole six-month follow-up period, allowing ≤5 cigarettes in total and verified by an eCO of ≤10 ppm [9]. Two further online, cross-sectional cohort studies also indicated an effective role of e-cigarettes for smoking cessation [3, 18]. In one survey among the first-time e-cigarette buyers intending to quit, 31% had abstained completely from smoking at 6 months, while in the second study, 50% of current smokers stated that e-cigarettes had ‘very much’ helped them to quit while only 4% stated that it did not help. Those are similar to findings of an earlier study by Etter et al investigating similar endpoints [17]. Those findings collectively demonstrate that when compared to existing smoking cessation aids, e-cigarettes perform comparably and may well have an effective role in smoking cessation and tobacco harm reduction.

E-cigarettes have also been shown to help reducing the number of cigarettes smoked by users who were unable to quit completely, or who were not intending to quit [17, 18, 28]. In one study, 23% of e-cigarette users reduced the number of cigarettes they smoked by more than half at 3 months [28]. In another study 67% claimed that they had reduced the number of cigarettes per day to some degree [18]. In the third study, 92% of former smokers said that e-cigarettes helped them reducing their daily cigarette consumption [17].

One of the controversial issues surrounding e-cigarettes’ effectiveness in smoking cessation is whether dual use of the devices along with tobacco cigarettes helps attenuate a nicotine addiction, or whether it only serves to perpetuate it [9, 18, 28]. On the issue of dual use of e-cigarettes and traditional cigarettes, Bullen et al found that smokers who intended to quit smoking but relapsed and continued using e-cigarettes had a significant reduction of tobacco cigarette consumption, leading them to believe that, just as dual use of nicotine replacement therapy and cigarettes is known to promote subsequent quit attempts, e-cigarettes may fulfil the same role [9]. Another study found that dual use of the two products by smokers, not necessarily intending to quit, resulted in a 31% abstinence rate at 6 months, suggesting that e-cigarettes could indeed act to attenuate nicotine addiction rather than perpetuate it [18]. On the other hand, Caponnetto et al found that dual users of e-cigarettes and tobacco who successfully quit were more likely to relapse, whereas those using e-cigarettes exclusively were more likely to remain abstinent [28]. Similarly, Lee et al found that adolescents who tried to quit smoking were more likely to use e-cigarettes but less likely to abstain entirely, suggesting that even if the devices do not promote a complete quit attempt, they may result in harm reduction by reducing the number of cigarettes smoked [27].

### Regulation

Regulatory authorities are in the process of reviewing the limited evidence available on e-cigarettes in order to make decisions on legislation, which will strongly influence the development of the e-cigarette market. In the US, the Food and Drug Administration (FDA) attempted initially to regulate e-cigarettes as drug-delivery devices [34]. However, this was blocked by lawmakers because the products made no therapeutic claim, arguing they should instead be regulated as tobacco products because they contained tobacco-derived nicotine [15]. Consequently, the FDA is now planning to regulate e-cigarettes as tobacco products, with limitations on online sales, sales to minors and advertising as well as imposing manufacturing quality control standards. The FDA released an amended rule encompassing e-cigarettes for public comment in Autumn 2013. During that period of regulatory wrangling, the absence of limitations facilitated e-cigarette companies to become a $2 billion industry in US [6].

The issue of the source of nicotine was raised in that debate because synthetic and tobacco plant-derived nicotine are treated differently from a legal perspective. Using the example of the US, products containing synthetic nicotine are either regulated as pharmaceutical products (and thus subject to the same standards required for therapeutic drug approval), or banned from the market if these standards are not met. On the other hand, products containing tobacco plant-derived nicotine and making no therapeutic claim are regulated as tobacco products and subject to the same standards as tobacco cigarettes. This legal nuance further complicates the regulatory debate about e-cigarettes. Since the source of nicotine in the devices is not always clear, this again emphasizes the need for further research into the contents, safety and manufacturing standards of e-cigarettes in order to properly inform regulatory decisions [15].

Commentary on the regulation of e-cigarettes has been underway in the US, Australia and Europe, where two main schools of thought have emerged. A pro-regulation group cites concerns including safety, a potential role as bridging and gateway products, and creating another income source for the tobacco industry as reasons for stringent regulation, possibly as therapeutic or drug-delivery devices [10, 15, 24, 33, 35]. One group of researchers argues that e-cigarettes have a net negative public health impact because they have not been shown to be better than NRT and pose significant risks in terms of safety and bridging use [33]. They also argue that smoking cessation aids such as NRT have not created the current smoking cessation trend, rather it has been well-organized government publicity campaigns that have de-normalized and de-glamorized smoking [33]. While this seems heavy handed, the argument pertaining to net public health impact is pertinent given safety concerns and the prevalence of recreational use [9].

Another point of contention for the pro-regulation group is that e-cigarettes are yet another tobacco industry marketing ploy. Rather than a strategy designed to offset declining tobacco sales, public health researchers argue e-cigarettes are a vehicle to addict future tobacco consumers, as well as creating a new income stream in the meantime. One in particular argues that by using e-cigarettes in this way, the tobacco industry aims to negate the current cessation trend, re-glamorize tobacco and smoking, present nicotine as benign to younger people and re-addict ex-smokers [10].

A less stringent approach is favored by a compromise-regulation group, which argues that premature over-regulation may extinguish a potentially beneficial product before concerns over its harms have been validated or its benefits disproven. These researchers contend that e-cigarettes should instead be regulated as tobacco products, subject to the same conditions regarding safety and manufacturing standards, as well as laws regarding sales to minors and advertising, and refute the arguments of the pro-regulation researchers in a number of ways [8, 24, 25, 36].

It is argued that e-cigarettes are not a big tobacco marketing ploy, and that on the contrary, laws banning nicotine products that aren’t tobacco or approved therapeutic products actually protect the tobacco industry’s dominant market position - a dominance that e-cigarettes may break if they are not regulated out of the market [8]. Over-regulation may also inhibit further product sophistication and innovation, stunting consumer uptake and reducing the potential of e-cigarettes as smoking cessation aid [24]. This group of researchers acknowledges that safety concerns are legitimate, but argues there is not yet any firm evidence to validate them, and consequently a great deal more research is required. On the issue of e-cigarettes as bridging and gateway products, it is conceded that aggressive marketing from big tobacco and other manufacturers may very well facilitate this type of undesirable uptake, and as such they should be subject to the same regulations as tobacco in terms of marketing and advertising [25]. Some of these sentiments may be hazardous, however, particularly regarding safety whereby it is suggested an untested product should be permitted to continue to proliferate unchecked, rather than the status quo whereby a new product is restricted until proven safe.

Reflecting the compromise-regulation approach, the UK government has already approved legislation to have all e-cigarettes regulated as medicines from 2016, with smokers advised to use traditional nicotine replacement therapies instead. Incongruously, this means that the tobacco companies, which already control a considerable amount of e-cigarette production, may eventually supply therapeutic products to the National Health Service (NHS). Indeed, the tobacco industry already does this with some NRT products, a situation which has been criticized for leading to their influence in tobacco policy decision-making by asserting itself as a producer of therapeutics [37]. Regulatory developments in the EU have progressed independently with a hybrid approach; in February 2014, the European Parliament voted to regulate e-cigarettes as tobacco products but those claiming therapeutic benefit as medicinal devices. This legislation will include a restriction on purchase age to a minimum of 18, close limitations on advertising and marketing including health warnings on packaging and the imposition of manufacturing standards [38]. Elsewhere, Brazil, Norway and Singapore, have banned the products altogether [6].

In Australia, the regulatory process has not been subject to the same debate, because the Therapeutic Goods Administration (TGA) essentially banned e-cigarettes at the outset [39]. Legislative discussions have thus been bypassed in favor of safety and control, presumably until further evidence of their potential harms and/or benefits becomes available. The TGA prohibits the importation, supply and sale of goods claiming therapeutic benefit that it has not approved, which applies to e-cigarettes marketed as smoking cessation aids. To cover remaining products, it also bans the sale of goods not containing tobacco that are designed to resemble tobacco products, whether the resemblance is in the product itself or its packaging. However, anecdotal evidence suggests that e-cigarettes with different flavors are easily available at different retail shops, even in $2 shops in Australia. That warrants further investigation for effective implementation of the ban in reality.

## Discussion

The most important finding of this review is that the long term health effects of e-cigarettes are unknown. The review further revealed that the substantial majority of e-cigarette users are middle-aged, predominantly male and current smokers. In one study, university students using e-cigarettes were an exception to this trend in that while nearly three quarters were former or current smokers, 12% had not previously smoked compared to other groups. However, there is some evidence of use amongst young people and non-smokers. Reasons for using e-cigarettes are mixed, with most people using them as smoking cessation aids but a significant proportion is using for recreational use. E-cigarettes have demonstrated quit and reduction rates comparable to existing NRTs, although results have been varied and further studies are recommended. Their ability to mitigate withdrawal symptoms, lack of side effects and capacity to closely simulate behavioral and handling process of smoking tobacco cigarettes, appear to benefit users. There is limited evidence to suggest that e-cigarettes are acting as ‘gateway’ products to introduce non-smokers to smoking. However, there is lack of data on e-cigarette use among adolescents and it is unknown whether the popularity of e-cigarettes has increased in this group.

Evidence regarding the health effects of e-cigarette use was mixed and warrants further investigations. At present, the balance of findings favors the non-toxicity of e-cigarettes’ vapor and e-cigarette use was not found to adversely affect lung function in the short term. Toxic substances have been found in e-cigarettes but at far lower levels than in traditional cigarettes. However, serious questions remain regarding their effect on important endpoints including cytotoxicity and long term health effects. This limited amount of safety research emphasizes the need for further investigation into the health effects of e-cigarettes, particularly the long term health effects.

Profiling users and usage patterns of e-cigarettes is an important first step in investigating the e-cigarette phenomenon and has major implications for public health programs and regulatory agendas. For example, evidence of use among people who have never smoked and adolescents gives weight to the bridging and gateway product concepts argued by several public health researchers [15, 24]. These terms refer to the phenomena of current smokers using e-cigarettes merely to perpetuate their habit in settings where smoking has been banned (bridging) and the initiation of nicotine and tobacco addictions amongst people who have never smoked (gateway) [15, 24]. However the bridging product may indeed help to reduce daily cigarette use, so should not be viewed unfavorably. Apart from the very small number of university students using e-cigarettes, who never smoked before, e-cigarettes did not appear to be acting as gateway products. The prevalence of recreational use of e-cigarettes gives some credence to the argument of one public health expert that e-cigarettes may be “re-glamorising” smoking amongst vulnerable population groups [10, 24].

E-cigarette’s potential as smoking cessation aids could tip the risk-benefit ratio in their favor. If scientific evidence proves that that they are effective in smoking cessation - and on the proviso that safety concerns are properly addressed through further investigations - the public health value of e-cigarettes may yet prove to be substantial. One of the controversial issues surrounding e-cigarettes’ effectiveness in smoking cessation is whether dual use of the devices along with tobacco cigarettes helps attenuate a nicotine addiction, or whether it only serves to perpetuate it [9, 18, 28]. Results relating to the dual use of e-cigarettes and traditional cigarettes assisting in abstinence and reduction in smoking have led some researchers to conclude that, just as dual use of NRT and cigarettes is known to promote subsequent quit attempts, e-cigarettes may fulfil the same role. This also appears to suggest that e-cigarettes may attenuate nicotine addiction rather than perpetuate it [18].

The studies we reviewed which investigated e-cigarettes for smoking cessation demonstrated that they could be effective in aiding quit attempts and reducing the number of cigarettes smoked [3, 9, 17, 18, 28]. Variability in rates of smoking cessation and reduction between studies may reflect differing levels of nicotine in e-cigarettes of different brands and batches, as well as variability in the amount of nicotine extracted by different users based on nuances of usage technique [40]. The study by Goniewicz et al. compared vapors of sixteen e-cigarette brands/models which were chosen based on their popularity in Polish, UK and US markets. Analyses showed that total nicotine in the vapor produced by a given series of puffs varied from 0.5–15.4 mg. The study also found that, on average, only 50-60% of the nicotine contained in each cartridge was actually vaporized [40].

Several plausible explanations for e-cigarettes’ apparent effectiveness in aiding smoking cessation and reduction have been elucidated [3, 17, 28]. First, they may mitigate withdrawal symptoms, which is a highly valuable effect given that overcoming withdrawal symptoms are known to be centrally implicated in a smoker’s ability to achieve and maintain abstinence [3]. Secondly, e-cigarettes have not at this stage been associated with significant side effects [3, 31]. Thirdly, studies suggest that there may be a behavioral component to the devices’ apparent effectiveness in aiding smoking cessation; physical handling and manipulation of a similar device, and the ability to respond to conditioned smoking cues, may be factors in attenuating craving for tobacco cigarettes [17, 28]. Studies found that when comparing nicotine and non-nicotine e-cigarettes both were equally effective in reducing tobacco cigarette consumption though those with nicotine performed better in terms of attenuating withdrawal symptoms, reinforcing the likelihood that this behavioral component may be a significant factor [17, 28].

E-cigarettes may, therefore, be able to either perpetuate or attenuate nicotine addiction, depending on whether users are motivated to quit or just use them recreationally [14, 21, 23, 35]. E-cigarettes may be an effective smoking cessation aid for those intending to quit smoking but also be used as bridging products which perpetuate users’ addiction to tobacco. It is this aspect of e-cigarette use that is of growing concern from a public health perspective and has serious implications for the regulation of e-cigarettes [10, 24, 36].

Findings from this review regarding user profiles and effectiveness as cessation aids should be used to inform regulatory decisions determining the future of e-cigarettes. Current debate about their regulation falls into two paradigms. One argues for strict regulation, similar to the way therapeutic products are governed. The other puts forward a compromised approach that ensures users’ safety while permitting commercial availability and ongoing product sophistication while their potential harms and benefits are further investigated [8, 10]. The argument for compromised regulation is based on the assumption that e-cigarettes currently have a net positive public health impact [8]. Given that some e-cigarette use is recreational and safety concerns persist, it can be argued that e-cigarettes do not yet have a net positive public health impact. It is possible that e-cigarettes are initiating or perpetuating more nicotine addictions than they are attenuating. Therefore, a regulatory agenda which controls those negative use trends while permitting use for cessation, appears to make sense. Regulation then needs to control the drivers of negative use (i.e. non-quit related recreational use), such as low cost, widespread availability and unfettered marketing [9, 12, 13, 25]. Since sales of e-cigarettes have been shown to be sensitive to price changes, policies increasing e-cigarette retail prices (such as limiting rebates, discounts and coupons and imposing tax on e-cigarettes), could potentially lead to significant reductions in e-cigarette sales [41].

Currently e-cigarettes are thought to be more expensive than tobacco in developing countries and so are viewed as more of a luxury product [42]. Two scenarios cause concern in this setting. Firstly, if e-cigarettes are shown to act as gateway products and their prices subsequently rise, they may promote further tobacco smoking initiation once e-cigarettes become unaffordable, as research shows price is a key factor in tobacco product initiation in developing countries [43]. Secondly, if e-cigarettes are shown to be safe alternatives to smoking that do not promote subsequent tobacco cigarette use, their higher prices may act as a disincentive for tobacco smokers to switch to a safer alternative. However, these are speculative scenarios and only highlight the need for further investigation into the use of the devices and pricing in developing countries.

### Limitations

Similar to other narrative reviews, this review had a number of limitations. Studies were selected to provide a comprehensive overview on the issues relating to e-cigarettes focusing on our objectives. As it was not a systematic review paper, it is possible that some key studies have been missed and selection biasness cannot be avoided. We tried to minimize these issues by following a specific search strategy unlike other narrative reviews. Quality of the selected studies was not assessed as that was beyond the scope of the review.

## Conclusions

Our overview of the literature on e-cigarettes has illuminated key areas of interest including their patterns of use, health effects, effectiveness for smoking cessation and regulatory issues. The cytotoxicity and long term health effects of e-cigarettes are unknown. Nevertheless the e-cigarette market continues to expand, largely driven by middle-aged smokers who claim to be using e-cigarettes in an attempt to reduce or quit smoking. E-cigarettes may have some potential as smoking cessation aids and, in the researchers’ view, should therefore be subject to further research and regulation similar to other nicotine replacement therapies.

## Authors’ information

MAR is a public health specialist and a physician, who is working as a Senior Research Fellow at The Cardiovascular Research Centre (CvRC) and St Vincent’s Centre for Nursing Research (SVCNR), Australian Catholic University Melbourne. He has a strong track record on tobacco research, both in developed and developing countries, specifically focusing on epidemiology. NH is a final year MD student from the University of Melbourne and worked at CvRC on this project as part of his ‘scholarly selective’ research project component of his course. AW is a cardiologist at St Vincent’s Hospital Melbourne, and Principal Research Fellow & Reader, Department of Medicine at the University of Melbourne. LWC is Professor of cardiovascular nursing and Director of CvRC and SVCNR. She is a nurse and clinical health researcher with over 20 years’ experience in cardiovascular health and research. Her other research expertise includes women and cardiovascular disease, cardiovascular risk assessment and prevention, as well as models of care around cardiovascular diseases.

## Abbreviations

E-cigarettes: Electronic cigarettes
NRT: Nicotine replacement therapy
FDA: United States Food and Drug Administration
TGA: Australian Government Therapeutic Goods Administration.

## References

[1] Foulds J, Veldheer S, Berg A (2011). Electronic cigarettes (e-cigs): views of aficionados and clinical/public health perspectives. Int J Clin Pract.

[2] Riker CA, Lee K, Darville A, Hahn EJ (2012). E-cigarettes: promise or peril?. Nurs Clin North Am.

[3] Dawkins L, Turner J, Roberts A, Soar K (2013). ‘Vaping’ profiles and preferences: an online survey of electronic cigarette users. Addiction.

[4] Romagna G, Allifranchini E, Bocchietto E, Todeschi S, Esposito M, Farsalinos KE (2013). Cytotoxicity evaluation of electronic cigarette vapor extract on cultured mammalian fibroblasts (ClearStream-LIFE): comparison with tobacco cigarette smoke extract. Inhal Toxicol.

[5] Noel JK, Rees VW, Connolly GN (2011). Electronic cigarettes: a new ‘tobacco’ industry?. Tob Control.

[6] Bialous SA, Sarma L (2014). Electronic cigarettes and smoking cessation: a quandary?. Lancet.

[7] Varkey B (2014). Electronic cigarettes: the good, the bad and the unknown. Curr Opin Pulm Med.

[8] Etter JF (2013). Should electronic cigarettes be as freely available as tobacco? Yes. BMJ.

[9] Bullen C, Howe C, Laugesen M, McRobbie H, Parag V, Williman J, Walker N (2013). Electronic cigarettes for smoking cessation: a randomised controlled trial. Lancet.

[10] Chapman S (2013). Should electronic cigarettes be as freely available as tobacco cigarettes? No. BMJ.

[11] Etter JF (2014). Commentary on Goniewicz et al. (2014): if wisely regulated, electronic cigarettes can make cigarettes obsolete. Addiction.

[12] Goniewicz ML, Knysak J, Gawron M, Kosmider L, Sobczak A, Kurek J, Prokopowicz A, Jablonska-Czapla M, Rosik-Dulewska C, Havel C, Jacob P, Benowitz N (2014). Levels of selected carcinogens and toxicants in vapor from electronic cigarettes. Tob Control.

[13] McAuley TR, Hopke PK, Zhao J, Babaian S (2012). Comparison of the effects of e-cigarette vapor and cigarette smoke on indoor air quality. Inhal Toxicol.

[14] Schivo M, Avdalovic MV, Murin S (2014). Non-cigarette tobacco and the lung. Clin Rev Allergy Immunol.

[15] Cobb NK, Abrams DB (2011). E-cigarette or drug-delivery device? Regulating novel nicotine products. New Engl J Med.

[16] Dutra LM, Glantz SA (2014). Electronic cigarettes and conventional cigarette use among U.S. adolescents: a cross-sectional study. JAMA Pediatr.

[17] Etter JF, Bullen C (2011). Electronic cigarette: users profile, utilization, satisfaction and perceived efficacy. Addiction.

[18] Siegel MB, Tanwar KL, Wood KS (2011). Electronic cigarettes as a smoking-cessation: tool results from an online survey. Am J Prev Med.

[19] Cho JH, Shin E, Moon SS (2011). Electronic-cigarette smoking experience among adolescents. J Adolesc Health.

[20] King BA, Alam S, Promoff G, Arrazola R, Dube SR (2013). Awareness and ever-use of electronic cigarettes among U.S. adults, 2010-2011. Nicotine Tob Res.

[21] Pearson JL, Richardson A, Niaura RS, Vallone DM, Abrams DB (2012). e-Cigarette awareness, use, and harm perceptions in US adults. Am J Public Health.

[22] Pepper JK, Reiter PL, McRee AL, Cameron LD, Gilkey MB, Brewer NT (2013). Adolescent males’ awareness of and willingness to try electronic cigarettes. J Adolesc Health.

[23] Sutfin EL, McCoy TP, Morrell HE, Hoeppner BB, Wolfson M (2013). Electronic cigarette use by college students. Drug Alcohol Depend.

[24] Cressey D (2013). Regulation stacks up for e-cigarettes. Nature.

[25] Borland R (2011). Electronic cigarettes as a method of tobacco control. BMJ.

[26] Goniewicz ML, Zielinska-Danch W (2012). Electronic cigarette use among teenagers and young adults in Poland. Pediatrics.

[27] Lee S, Grana RA, Glantz SA (2014). Electronic cigarette use among Korean adolescents: a cross-sectional study of market penetration, dual use, and relationship to quit attemptsand former smoking. J Adolesc Health.

[28] Caponnetto P, Campagna D, Cibella F, Morjaria JB, Caruso M, Russo C, Polosa R (2013). EffiCiency and Safety of an eLectronic cigAreTte (ECLAT) as tobacco cigarettes substitute: a prospective 12-month randomized control design study. PLoS One.

[29] Chorti M, Poulianiti K, Jamurtas A, Kostikas K, Tzatzarakis M, Vynias D, Koutedakis Y, Flouris A, Tsatsakis A (2012). Effects of active and passive electronic and tobacco cigarette smoking on lung function. Toxicol Lett.

[30] Flouris AD, Chorti MS, Poulianiti KP, Jamurtas AZ, Kostikas K, Tzatzarakis MN, Wallace HA, Tsatsakis AM, Koutedakis Y (2013). Acute impact of active and passive electronic cigarette smoking on serum cotinine and lung function. Inhal Toxicol.

[31] Vardavas CI, Anagnostopoulos N, Kougias M, Evangelopoulou V, Connolly GN, Behrakis PK (2012). Short-term pulmonary effects of using an electronic cigarette: impact on respiratory flow resistance, impedance, and exhaled nitric oxide. Chest.

[32] Bahl V, Lin S, Xu N, Davis B, Wang YH, Talbot P (2012). Comparison of electronic cigarette refill fluid cytotoxicity using embryonic and adult models. Reprod Toxicol.

[33] Stanbrook MB (2013). Regulate e-cigarettes as drug-delivery devices. CMAJ.

[34] Office of Information and Regulatory Affairs US Government (2013). “Tobacco Products” Subject to the Federal Food, Drug, and Cosmetic Act, as Amended by the Family Smoking Prevention and Tobacco Control Act.

[35] Kuehn BM (2009). FDA: electronic cigarettes may be risky. JAMA.

[36] Benowitz NL, Goniewicz ML (2013). The regulatory challenge of electronic cigarettes. JAMA.

[37] Torjesen I (2013). E-cigarettes are to be regulated as medicines from 2016. BMJ.

[38] European Commission (2014). Questions & Answers: New rules for tobacco products.

[39] Department of Health Australian Government (2013). Electronic Cigarettes.

[40] Goniewicz ML, Kuma T, Gawron M, Knysak J, Kosmider L (2013). Nicotine levels in electronic cigarettes. Nicotine Tob Res.

[41] Huang J, Tauras J, Chaloupka FJ (2014). The impact of price and tobacco control policies on the demand for electronic nicotine delivery systems. Tob Control.

[42] Kostova D, Ross H, Blecher E, Markowitz S (2011). Is youth smoking responsive to cigarette prices? Evidence from low- and middle-income countries. Tob Control.

[43] Kostova D, Chaloupka FJ, Shang C (2014). A duration analysis of the role of cigarette prices on smoking initiation and cessation in developing countries. Eur J Health Econ.

